# The Activation of the Mirror Neuron System during Action Observation and Action Execution with Mirror Visual Feedback in Stroke: A Systematic Review

**DOI:** 10.1155/2018/2321045

**Published:** 2018-04-24

**Authors:** Jack J. Q. Zhang, Kenneth N. K. Fong, Nandana Welage, Karen P. Y. Liu

**Affiliations:** ^1^Department of Rehabilitation Sciences, The Hong Kong Polytechnic University, Kowloon, Hong Kong; ^2^School of Science and Health, Western Sydney University, Penrith, NSW, Australia

## Abstract

**Objective:**

To evaluate the concurrent and training effects of action observation (AO) and action execution with mirror visual feedback (MVF) on the activation of the mirror neuron system (MNS) and its relationship with the activation of the motor cortex in stroke individuals.

**Methods:**

A literature search using CINAHL, PubMed, PsycINFO, Medline, Web of Science, and SCOPUS to find relevant studies was performed.

**Results:**

A total of 19 articles were included. Two functional magnetic resonance imaging (fMRI) studies reported that MVF could activate the ipsilesional primary motor cortex as well as the MNS in stroke individuals, whereas two other fMRI studies found that the MNS was not activated by MVF in stroke individuals. Two clinical trials reported that long-term action execution with MVF induced a shift of activation toward the ipsilesional hemisphere. Five fMRI studies showed that AO activated the MNS, of which, three found the activation of movement-related areas. Five electroencephalography (EEG) studies demonstrated that AO or MVF enhanced mu suppression over the sensorimotor cortex.

**Conclusions:**

MVF may contribute to stroke recovery by revising the interhemispheric imbalance caused by stroke due to the activation of the MNS. AO may also promote motor relearning in stroke individuals by activating the MNS and motor cortex.

## 1. Introduction

Stroke is one of the leading causes of adult disability. Patients commonly suffer lasting motor impairments and functional disability following a stroke [1]. A substantial number of advanced rehabilitation strategies have been applied in upper limb stroke rehabilitation, such as robot-assisted therapy [2], constraint-induced movement training (CIMT) [3], and virtual reality- (VR-) based rehabilitation [4], which are aimed at helping stroke survivors relearn motor skills through intensive training. These rehabilitation strategies have been reported to improve patients' motor functions by inducing experience-dependent neuroplasticity in their damaged hemispheres [5–7]. However, the neuroplasticity resulting from intensive-based interventions may be limited if the residual motor functions of the patients are also extremely limited. It is crucial to find an adjunct therapy, on top of limb training, to enhance the recovery of the ipsilesional motor cortex of patients with severe hemiplegia, in order to overcome the learned nonuse phenomenon in such patient populations [8].

There is evidence to support the theory that cortical areas involved in motor execution can be activated by observing actions performed by others, which is attributed to the function of the mirror neuron system (MNS). The MNS is a class of neural substrates that discharges during action observation (AO) and action execution [9, 10]. The MNS is also associated with various human functions, such as motor preparation [11], motion imitation [12, 13], language [14], and emotion recognition [15, 16]. In humans, the core MNS is understood to be located in the inferior frontal gyrus (IFG), including the ventral premotor cortex (PMv), the inferior parietal lobule (IPL), and the intraparietal sulcus (IPS) [9, 17]. An extended MNS involves additional brain regions, such as the primary motor cortex, the primary somatosensory cortex, and the middle frontal cortex [18]. A bilaterally distributed parietofrontal network with mirror neuron (MN) properties (i.e., parietofrontal MNS) has been proposed, which serves as a neural substrate to achieve the transformation of visual information into cortical areas for motor execution (i.e., visuomotor transformation) [19].

Based on this theory, researchers believe that the motor cortex could be primed by activating the MNS, thus boosting the efficacy of standardized rehabilitation for patients after strokes [17, 20]. Subsequently, various rehabilitative strategies, aimed at facilitating the motor cortex through activating the MNS, have been applied in stroke rehabilitation, including action observation training (AOT) [21, 22] and action execution with MVF [23]. AOT usually consists of a session of AO followed by a session of imitating the observed action [22]. Some clinical trials have supported the efficacy of AO as a motor priming tool in stroke rehabilitation [24–28]. Previous neuroimaging studies have identified a bilateral AO network over the frontal, parietal, temporal, and occipital areas in the brain, which encompass the core MNS [29, 30]. Action execution with MVF, including mirror therapy (MT) [23], mirror box therapy [31], and VR-based MT [32], is already a commonly employed regimen in stroke rehabilitation. By virtue of MVF, patients receive a visual illusion showing that their hemiplegic upper limbs are moving normally when they move their nonparetic upper limbs simultaneously [23]. MVF could boost the effects of the conventional upper limb rehabilitation of stroke [33]. It has been proposed that the training-induced effects of MVF arise from the activation of the ipsilesional primary motor cortex by enriching the visual and proprioceptive inputs to the MNS [10, 19, 34], but this hypothesis has not been duly confirmed in human studies [35].

It is feasible nowadays for researchers to objectively measure the brain's activities before and after interventions using advanced functional neuroimaging and electrophysiological techniques [36]. An increasing number of studies regarding the effects of these two promising motor priming techniques (AO and MVF) on brain activation in stroke individuals have been published. However, there is a lack of focused reviews investigating the effects of AO or action execution with MVF on the activation of MNS and its subsequent effects on the activation of the motor cortex in patients who have had a stroke. We conducted this systematic review to evaluate the concurrent and training effects of AO and action execution with MVF on the activation of the MNS in stroke individuals, by reviewing available experimental studies as well as clinical trials with functional neuroimaging or electrophysiological examinations. In order to understand the role of the MNS in upper limb stroke rehabilitation, we have summarized the following information in this review: (1) MNS activation and (2) MNS activation and its relationship with the activation of the motor cortex.

## 2. Methods

### 2.1. Literature Search

A literature search for relevant studies was conducted using CINAHL (the Cumulative Index to Nursing and Allied Health Literature), PubMed, PsycINFO, Medline, SCIE (Science Citation Index Expanded), and SCOPUS. Two of the authors of this review independently identified the relevant studies. Keywords used during the search were “stroke” OR “hemiplegia”; “action observation” OR “action observation training” OR “mirror visual feedback” OR “mirror neuron” OR “mirror therapy” OR “mirror box therapy”; and “functional imaging” OR “functional magnetic resonance imaging” OR “fMRI” OR “electroencephalography” OR “EEG” OR “near-infrared spectrometry” OR “NIRS” OR “magnetoencephalography” OR “MEG” OR “positron emission tomography” OR “PET”. The date of publication was limited to 10 years, from January 2007 to November 2017. The reference lists of the retrieved articles were manually searched to identify any further relevant articles.

### 2.2. Selection Criteria

We used the PICOS method to formulate our selection criteria. Studies that satisfied all the following criteria were considered for this review.

*Population (P)*: studies recruiting adult patients diagnosed with having had strokes; *intervention (I)*: interventions or experimental paradigms using AO or MVF in regard to upper limb actions; *comparison (C)*: control conditions without AO or MVF or using sham AO or MVF; *outcomes (O)*: studies providing anatomical evidence of brain activation induced by AO or action execution with MVF, as represented by signal changes in PET, fMRI, or fNIRS; or using previously validated electrophysiological indices of MN activities, such as event-related desynchronization (ERD) of the mu band (i.e., mu suppression) [37] or the ERD of the beta band (i.e., beta suppression) [38]; or employing an advance analysis to explore the neural network related to AO or MVF, including but not limited to dynamic casual modelling (DCM) in regard to fMRI or a coherence analysis of EEG; and *study design (S)*: clinical trials investigating the training effects or experimental studies investigating the concurrent effects of the relevant experimental condition.

Studies were excluded if (1) they only recruited healthy subjects or patients with other primary diagnoses (e.g., Parkinson's disease); (2) they only focused on the lower limb or trunk actions; (3) the final analyzed sample size was less than five; (4) they were published as conference proceedings, dissertations, or in books; and (5) they were not published in English language.

### 2.3. Data Extraction

After identifying relevant studies, two authors independently extracted the following information from each article: (1) the characteristics of participants; (2) the protocol of the intervention or experiment; (3) the modalities of the functional neuroimaging or electrophysiological techniques used in the study; and (4) the main results of the studies. Any disagreement was settled by discussion with the third author.

### 2.4. Quality Assessment

We assessed the quality of the randomized controlled trials (RCTs) in regard to the training effects of AOT or action execution with MVF in patients who have had strokes, based on the Physiotherapy Evidence Database (PEDro) scale. Both independent reviewers evaluated each article. The PEDro scale consists of 11 items. The first criterion, item eligibility, is not scored, as it is used as a component of external validity. The other criteria included random allocation, concealment of allocation, baseline equivalence, blinding procedure, intention to treat analysis, adequate follow-up, between-group statistical analysis, measurement of data variability, and point estimates. Any scoring discrepancies were resolved.

## 3. Results

### 3.1. Identification Process for the Selection of the Studies

**Figure 1:**
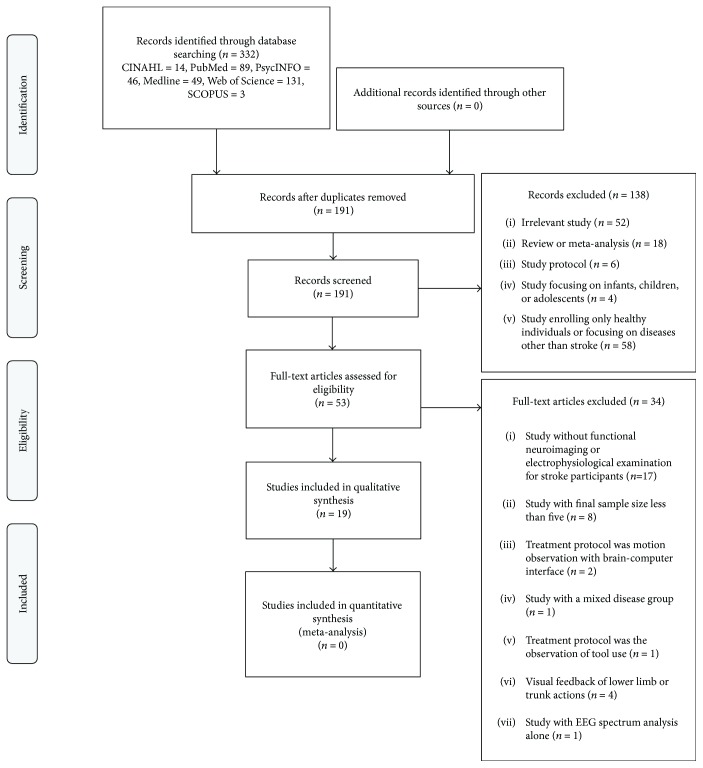
Flowchart of literature search.

The initial search yielded 332 results. After removing duplicates, a total of 191 records were screened, of which, 138 citations were excluded for the following reasons: the studies were reviews or meta-analyses (*n* = 18); the studies' protocols (*n* = 6); the studies focused on infants, children, or adolescents (*n* = 4); the studies enrolled only healthy participants or patients with neurological diseases other than strokes (*n* = 58); or the studies were irrelevant (*n* = 52). The remaining 53 articles were subjected to full-text reading, of which 34 articles were removed for the following reasons: the studies did not use functional neuroimaging or electrophysiological techniques for stroke participants (*n* = 17); the final analyzed sample sizes of the studies were less than five (*n* = 8); studies of motion observation with a brain-computer interface (*n* = 2); the visual feedback was based on the lower limbs or trunk actions, rather than upper limb actions (*n* = 4) [39–42]; or the studies used EEG spectrum analysis alone (*n* = 1) [43]. One study focused on the functional neuroplasticity induced by observing the skills of tool use, which was hard to compare with other protocols of AO. Another study enrolled both patients who have had strokes and brain tumors, and the data of stroke participants could not be separated; these studies were hence excluded [16, 44]. Finally, 19 articles satisfied our inclusion criteria and were included in the present review [13, 22, 28, 34, 45–59].  Figure 1 shows the identification process for the selection of studies.

### 3.2. Clinical Trials regarding the Training Effects of Long-Term Intervention

Among the included studies, six studies focused on the training effects of long-term therapeutic programs [22, 28, 45, 46, 52, 59]. Four of these were RCTs [22, 28, 45, 52], and the other two were interventional studies with pre-post comparisons [46, 59]. Three studies investigated the training effects of bimanual training with MVF (four- to eight-week interventions) [45, 46, 52]. Of these, two identified a shift of activation toward the ipsilesional hemisphere [46, 52], evidenced by fMRI. Cortical areas activated by MVF mainly included the primary motor cortex [46, 52] and the premotor cortex (PMC) [46]. A study with EEG reported that mu suppression over sensorimotor cortex (SMC) was higher in the group with MVF [45]. Sun et al., who also used mu suppression as an index, reported additional benefits of AO on the basis of motor imagery (MI) in regard to enhancing mu suppression over the ipsilesional SMC, compared with the control group (MI alone) [28]. A fNIRS study measured the difference in brain activity of participants before and after four weeks of MT in addition to conventional rehabilitation; however, the difference in the activation pattern over the primary motor cortex and the precuneus was insignificant over time [59].

An RCT investigating the training effects of AO found that the four-week AOT (AO followed by imitation) induced more evident activation over bilateral PMv, bilateral superior temporal gyrus (STG), supplementary motor area (SMA) over the contralesional hemisphere, and supramarginal gyrus (SMG) over the ipsilesional hemisphere, relative to the control group watching nonbiological videos followed by action execution [22]. Characteristics of these studies are summarized in Table 1.

### 3.3. Experimental Studies with fMRI Findings

Eight articles explored the concurrent effects of AO or action execution with MVF on brain activation, evidenced by fMRI [34, 47, 48, 50, 53, 55–57]. In studies regarding MVF, Michielsen et al. found that bimanual movement with MVF led to significant activation of the precuneus and the posterior cingulate cortex (PCC), rather than the MNS [53]. However, Saleh et al. [34, 55] reported that the ipsilesional primary motor cortex was activated by MVF, and connectivity between the ipsilesional primary somatosensory cortex and the primary motor cortex was stronger, relative to the control group without MVF [55]. The source of the ipsilesional primary motor cortex activation was further found by the DCM to be the contralesional intraparietal sulcus (IPS) [34]. Wang et al. reported that lateralized activation toward the affected hemisphere was in favor of virtual MVF, as reflected by the peak *T* value of the precuneus in the majority of their samples [57].

For studies concerning AO, Szameitat et al. found that AO of wrist movement activated the PMC and IPL; however, the pattern of neural activation in action execution more resembled MI, rather than AO [56]. Garrison et al. reported that left IFG, SMG, and bilateral precentral gyrus were activated during right-hand (paretic side) observation of reach and grasp actions. Lateralized activation toward the ipsilesional hemisphere was also noted [50]. During left-hand AO (nonparetic side), the bilateral activation was relatively symmetrical in stroke individuals. Brunner et al. [47] devised a protocol to observe bimanual action; they reported that stroke individuals (within one-to-two weeks after the stroke) showed activation in inferior and superior parietal lobes, IFG, and the primary motor cortex during AO. In the second fMRI exam (three months after the stroke), the neural response to AO was extended to more movement-related areas, including the PMC, primary motor cortex, and the SMA. The neural response to AO was increased from one or two weeks to three months after the individual suffered a stroke. Dettmers et al. compared the brain activities during AO and MI of patients with left or right subcortical strokes and reported that patients with left subcortical strokes presented higher levels of activity than those with right subcortical strokes [48]. The brain activation induced by AO or MVF shown by fMRI are summarized in Table 2.

### 3.4. Experimental Studies with EEG or MEG

Four experiments measured the concurrent effects of AO on mu rhythm using EEG [13, 49, 51, 58]. Another study used MEG to measure the difference in beta suppression during bimanual movement with and without MVF [54]. Kuk et al. reported the whole brain topography based on mu rhythm before and after AO and found that the middle frontal gyrus (MFG) was less active after a total of five sessions of AO [51]. Frenkel-Toledo et al. reported that observations of the reach-and-grasp hand action could induce mu suppression over the SMC, but the magnitude of mu suppression was significantly lower in the affected hemisphere, relative to the unaffected hemisphere. Mu suppression over the unaffected side was attenuated in patients with lesions over the right IPL [13, 49]. Tani et al. also showed that AO of the open-and-close action by the paretic hand induced stronger mu suppression than the MI of the same action in stroke individuals [58]. Interhemispheric imbalance of movement-related beta suppression was noted in stroke participants in the study by Rossiter et al. when performing the bimanual open-and-close action. The initial asymmetricity was partially attenuated by MVF [54]. Characteristics of the experimental studies are summarized in Table 3, and brain activation induced by AO or MVF, measured by fMRI, is summarized in Table 2.

### 3.5. Methodological Quality of Included Randomized Controlled Trials

Four RCTs were included in this review [22, 28, 45, 52]. The results of the assessment of methodological quality are summarized in Table 4.

## 4. Discussion

The present study is aimed at systematically evaluating the evidence of MNS activation induced by AO or MVF and its potential effects on the activation of the motor cortex in patients who have had strokes. The main findings of the present review are (1) the ipsilesional primary motor cortex can be facilitated by MVF [46, 52], which may be achieved by recruiting the MNS [34, 55]; (2) long-term action execution with MVF resulted in a shifted activation toward the ipsilesional hemispheres in patients who have had strokes; hence, a more symmetrical state between the two hemispheres may be achieved [46, 52]; (3) AO induced broader brain activation in the frontal, parietal, temporal, and occipital areas in patients who have had strokes, which encompassed the MNS, as well as cortical areas of motor execution, including the primary motor cortex, PMC, and SMA [22, 47, 48, 50]; (4) mu suppression can be induced by AO in patients who have had strokes; however, mu suppression over the affected hemisphere is relatively diminished [13, 49, 58]; and (5) MVF [45] or AO [28] embedded in long-term rehabilitation could bring about additional neurophysiological effects in patients after they have had strokes, reflected by more evident mu suppression, which may indicate that MN activities can be increased by this training.

A classical pathological change following stroke involves the activities of the affected hemispheres being suppressed, while those of less affected hemispheres are heightened, due to interhemispheric competition rivalry [60]; hence, successful motor recovery in patients who have had strokes could be achieved by normalizing the interhemispheric asymmetricity and promoting the neuroplasticity of the ipsilesional motor cortex [61, 62]. As the present review has shown, long-term MVF can contribute to a shift in activation toward the affected hemisphere [46, 52]. Furthermore, MVF transiently attenuates the asymmetric activities of movement-related beta suppression [54]. These findings can partially explain the beneficial effects of MT, which induces more symmetrical activities between the two hemispheres in patients who have had strokes. This is in line with previous findings regarding the effects of MVF on the healthy brain [35]. However, evidence to support the way in which AO can induce a shift of activation toward the affected hemisphere is relatively limited [50].

The difference between the activation patterns of MVF and AO can be identified. MVF mainly activates the ipsilesional primary motor cortex [34, 40, 46, 52, 55], the PMC [46], the primary somatosensory cortex [34, 40, 55], and the IPL [34, 55]. Two articles (one study) using effective connectivity and DCM have proposed that MVF could increase the connectivity between the ipsilesional primary somatosensory and primary motor cortex [55]. This study also suggests that the activation of the ipsilesional primary motor cortex may arise from contralesional IPS [34], which is a part of the MNS. These results are in line with the assumed functions of the MNS: visuomotor transformation. However, this conclusion should be interpreted with caution, since some studies have not identified the MNS activation by MVF in either patients who have had strokes [53] or healthy subjects [35]. The studies by Saleh et al. did not choose another frontal MNS (e.g., PMC) as the node in the DCM [34, 55]. As the first fMRI study with DCM that supports the activation of the MNS induced by MVF and its subsequent effects on the activation of the primary motor cortex [34], it is worthwhile to further explore the neural network underlying the MVF, in order to explain the role of the MNS in the network.

For AO, the activated brain regions were much broader, including IFG [47, 50], the PMC [22, 47, 48], the IPL [47], the primary motor cortex [47, 50], and temporal and occipital structures, which encompass the parietofrontal MNS as well as the cortical areas for motor execution (e.g., the primary motor cortex, PMC, and SMA). Small et al. proposed a model of brain repair after a stroke, which hypothesized that the MNS activation induced by AO may promote the reorganization of the cortical motor loop (i.e., the primary motor cortex, PMC, and SMA), thereby improving the motor functions of stroke survivors [63]. Our findings provide anatomical evidence to support this model. Observations of the bimanual action elicited a similar activation pattern as the execution of the same action in one study [47], whereas another study showed that AO (of a simple wrist movement) activated a part of the PMC and IPL, which did not resemble the activation pattern of action execution to such a great extent [56]. This difference may be attributed to different experimental paradigms of AO. The AO network, which may be involved in the understanding of motor intention, may have a stronger response to an object-directed or goal-directed action [64, 65], relative to a single action without meaning, although this is still inconclusive [66]. All in all, the results are still consistent with previously defined bilateral AO networks in healthy human brains [29, 30]. The neural network underlying the AO remains unclear.

The activation patterns of MVF and AO are obviously different. AO elicited broader activation of the frontal, parietal, temporal, and occipital areas, while the activated regions of MVF mainly covered the frontal and parietal structures. The difference between these two regimens is that the participants were required to perform bilateral or unilateral movement themselves when observing the visual feedback in the MVF experiments, while this was not required in the AO experiments. Previous studies have assumed that there are different neural networks in response to AO and MVF [35, 67], and this opinion was confirmed by the present review in the stroke cohorts. Even though the underlying neural network cannot be fully understood at this stage, MNS activation seems to play a key role in both AO- and MVF-induced functional plasticities. There have been several TMS studies that have provided indirect evidence of the activation of the primary motor cortex by AO or MVF [27, 68]. Therefore, these two modalities can be viewed as optional motor priming tools for stroke rehabilitation. However, there is still a lack of studies directly comparing the clinical improvements and the pattern of neuroplasticity induced by MVF and AO [69] and whether or not the activation pattern is congruent with the clinical improvements in patients with stroke.

Brain waveforms recorded by EEG are altered by AO, reflected by lower alpha power and higher beta power over the frontal, central, and occipital electrodes [41, 42]. Some studies have also shown that the electrophysiological responses to AO may decrease after repetitive stimulations [43, 51]. These changes may be related to the changes in cognitive activities related to the understanding of motor intention after receiving visual feedback [70]. However, pure spectrum analysis is less likely than detailed EEG analyses, for example, time-frequency analysis, to reflect MN activity and stroke recovery [71, 72]. A recent meta-analysis indicated that both AO and action execution could induce the suppression of mu rhythm with a significant effect size [73]. This property of dual activation makes the mu suppression a signature of human mirror neuron activity [37, 74]. Regarding the studies included in the present review, two articles investigated AO-induced mu suppression in stroke individuals and its relationship with brain lesions [13, 49]. They found that the magnitude of mu suppression was reduced in the affected hemisphere, relative to the unaffected hemisphere, which was also identified by another study [58]. Two RCTs demonstrated that AO- or MI-induced mu suppression can be enhanced after long-term AOT [28] or training with MVF [45], which implies enhanced MN activities after the AO or MI training. Lesion analysis also showed that the damage over IPL or IFG was correlated with the diminished mu suppression, which indicates that mu suppression may be a specific index of MN activities [13, 16, 49].

One study demonstrated that behavioral improvement is correlated with the neural response to AO (measured by fMRI) in stroke individuals, which indicates that the AO-induced neural response is likely to be an indicator that can evaluate the arm motor recovery of patients who have had strokes within the first three months [47]. As the MNS is correlated with motion imitation [75], its activation may be a neurobiomarker that can measure the potential of motor learning in patients who have had strokes [47], which could also be measured by mu suppression. The predictive value of mu suppression and its relationship with motor improvement remain speculative. Other frequency bands related to action execution and AO, such as the beta band, might also be suitable candidates in regard to measuring MN activities [38, 76], although evidence of this in the stroke population is fairly limited [54]. As EEG is a relatively low-cost technique, further studies are encouraged to longitudinally explore the role of the ERD of different frequency ranges over various brain regions in patients who have had strokes. This neurobiomarker may also serve as a useful reference of the patients' motor recovery trajectory and motor relearning potential, promoted by MNS activation in stroke individuals.

There are some limitations in the present review. First, the heterogeneous protocols of AO and MVF and different experimental designs in regard to implementing neuroimaging hindered us from giving a firm and precise conclusion. Second, potentially confounding factors cannot be fully explained, based on currently available evidence, such as the dominance of handedness [48] and the nature of stroke lesions [48], which may result in different responses to AO and MVF. Further studies are warranted to answer these questions. Finally, restricting our review to English publications may have resulted in language bias.

## 5. Conclusions

MVF may contribute to stroke recovery by revising the interhemispheric imbalance, and MNS recruitment may be one of the potential neural mechanisms in this process. AO is associated with the activation of the MNS and motor cortex, which may promote motor relearning in stroke individuals. More rigorous studies with functional neuroimaging or electrophysiological techniques should be performed to further explain the different functional neural networks underlying AO or MVF and to explore the relationship between MN activities and clinical recovery in patients who have had strokes.

## Figures and Tables

**Table 1:** Characteristics of studies regarding the effects of long-term training on brain activation in stroke patients.

Study	Characteristics of stroke participants	Severity of hemiplegia	Intervention protocol	Intervention protocol	Imaging modality	Main results	
						Neuroimaging findings	Behavioral improvements
Ertelt et al. [22]	15 chronic stroke patients (>six months after stroke), aged 38–69.	Moderate arm paralysis (FAT: 0 to 5; WMFT (time): 2.41 to 41.29 seconds).	EG (*n* = 7): AOT (videos of upper limb actions followed by practice of observed actions, using the paretic upper limb); CG (*n* = 8): control (nonbiological videos followed by practice of the same actions, using the paretic upper limb).	Four-week intervention (90 minutes/session, 18 sessions in total).	fMRI (EG = 7 and CG = 6).	More evident activation of PMv, SMA, insula, and STG over the nonaffected hemisphere and PMv, SMG, and STG over the affected hemisphere in EG than CG.	FAT posttreatment (EG > CG) WMFT-posttreatment (−) SIS-posttreatment (EG > CG). No significant difference between posttreatment and follow-up in three assessments.

Michielsen et al. [52]	40 chronic stroke patients (>one year after stroke), aged 55.3 ± 12.0 (MT)/58.7 ± 13.5 (CG).	Brunnstrom stage for the upper extremity III-V.	MT (*n* = 20): bimanual exercise with MVF of the unaffected hand; CG (*n* = 20): bimanual exercise with direct view of both hands.	Six-week intervention (one hour/session, five sessions/week).	fMRI (MT = 9 and CG = 7).	A shift of activation toward the M1 over the affected hemisphere in MT.	FMA posttreatment (MT > CG); FMA 6-month follow-up (−) Jamar handheld dynamometer (−) Tardieu scale (−) ARAT (−) Stroke-ULAM (−) EQ-5D (−).

Bhasin et al. [46]	20 stroke patients (three 14 months after stroke), aged 45.45 ± 6.6.	Brunnstrom stage of the hand II-IV.	Bilateral hand exercise with virtual MVF of the nonparetic hand.	Eight-week intervention (five days/week, 60–90 minutes/session).	fMRI.	LI of BA 4 and BA 6 was increased at eight weeks.	FM scale (posttreatment and 24-week follow-up > pretreatment) MBI (posttreatment and 24-week follow-up > pretreatment) MRC grade scale (−) Brunnstrom stage (−).

Bae et al. [45]	20 stroke patients (<six months after stroke), aged 55.2 ± 8.5 (MT)/52.6 ± 11.2 (CG).	Brunnstrom stage of the hand II–IV.	MT (*n* = 10): bilateral upper limb exercise with MVF; CG (*n* = 10): paretic arm exercise only.	Four-week intervention (30 minutes/session, five times/week).	EEG.	Mu suppression at C3, Cz, and C4 was higher in MT than CG.	MFT posttreatment (MT > CG).

Sun et al. [28]	10 stroke patients (<two months after stroke), aged 59.4 ± 4.94.	Severe arm paralysis (FMA: 10 to 25).	On top of the CR, patients received the training: EG (*n* = 5): AO (paretic upper limb actions) with MI; CG (*n* = 5): MI alone.	Four-week intervention.	EEG.	AO with MI increased to higher mu suppression over C3 than MI at week 2, 3 and 4.	FMA week 2, 3 and 4; (EG > CG) PST week 3 and 4 (EG > CG).

Brunetti et al. [59]	11 stroke patients (15–92 days after stroke), aged 49–74.	Severe hand paralysis (a wrist extension of less than 20 degrees and metacarpophalangeal joint extension of less than 10 degrees).	On top of the CR, patients performed a bilateral exercise with MVF of nonparetic side.	Four-week intervention (30 minutes/session, five sessions/week).	fNIRS.	The activation pattern of M1 and precuneus was stable over time.	Six of eleven patients showed improvement (gain scores from one to eight) in FMA-finger.

FAT: Frenchay arm test; WMFT: Wolf motor function test; EG: experimental group; CG: control group; AOT: action observation training; fMRI: functional magnetic resonance image; PMv: ventral premotor cortex; SMA: supplementary motor area; STG: superior temporal gyrus; SMG: supramarginal gyrus; SIS: stroke impact scale; MVF: mirror visual feedback; MT: mirror therapy; M1: primary motor cortex; FMA: Fugl-Meyer assessment; ARAT: action research arm test; Stroke-ULAM: stroke upper limb activity monitor; EQ-5D: EuroQOL five-dimension questionnaire; LI: laterality index; BA: Broadman area; MBI: modified Barthel index; MRC: Medical Research Council; EEG: electroencephalography; BA: Broadman area; MFT: manual function test; PT: physical training; CR: conventional rehabilitation; AO: action observation; MI: motor imagery; ERD: event-related desynchronization; PST: pinch strength test; fNIRS: functional near-infrared spectroscopy.

**Table 2:** Brain activation or lateralization of brain activation measured by fMRI.

Brain areas	Concurrent effect of MVF versus the control without MVF during a motor task	Long-term rehabilitation with MVF versus the control without MVF	Concurrent effect of AO versus the control	Long-term rehabilitation with AO versus the control without AO
*Frontal lobes*				
Primary motor cortex	Ipsilesional activation [34, 55]	Ipsilesional lateralization [46, 52]	Bilateral activation [47]; ipsilesional lateralization [50]	
Premotor cortex		Ipsilesional lateralization [46]	Bilateral activation [47]; ipsilesional activation [56]	Bilateral activation [22]
Supplementary motor area			Bilateral activation [47]	Contralesional activation [22]
Superior frontal gyrus			Ipsilesional activation [48]	
Inferior frontal gyrus			Bilateral activation [47]; ipsilesional lateralization [48, 50]	
Prefrontal gyrus			Ipsilesional activation [48]	

*Parietal lobes*				
Primary somatosensory cortex	Bilateral activation [34, 55]			
Superior parietal gyrus			Bilateral activation [47]; ipsilesional activation [48]	
Precuneus	Bilateral activation [34, 53, 55]; ipsilesional lateralization [57]			
Inferior parietal gyrus			Ipsilesional activation [56]; bilateral activation [47]	
Supramarginal gyrus	Contralesional activation [34, 55]		Ipsilesional lateralization [50]	Ipsilesional activation [22]
Intraparietal sulcus	Contralesional activation [34, 55]			
Posterior cingular cortex	Contralesional activation [53]			

*Temporal lobes*				
Superior temporal gyrus				Bilateral activation [22]
Inferior temporal gyrus			Bilateral activation [47]	

*Occipital lobes*				
Occipital gyrus			Bilateral activation [47]	

Notes: MVF: mirror visual feedback; AO: action observation. Garrison et al. [50]: results of AO of the paretic hand movement were used; Bhasin et al [46]: the result of within-group difference was used, because the study did not have a control group.

**Table 3:** Characteristics of studies regarding the concurrent effects of single-session or multiple-session experiments on brain activation in stroke patients.

Study	Characteristics of stroke participants	Severity of hemiplegia	Experiment conditions	Imaging modality	Main results
Garrison et al. [50]	12 chronic stroke patients (two to 17 years after stroke), aged 39 to 85.	Moderate to severe arm paresis (FMA-UE: 13 to 48).	AO (reach to grasp objects by the right or left hand) and fixation.	fMRI.	Right-hand (paretic side) AO resulted in lateralization toward the left hemisphere, including IFG pars opercularis, IFG pars triangularis, SMG, and precentral gyrus.

Michielsen et al. [53]	18 chronic stroke patients (>one year after stroke), aged 54.7 ± 9.9.	Brunnstrom stage for the upper extremity III-V.	Unimanual exercise (open-and-close action by the unaffected hand) with MVF; unimanual exercise without MVF; bimanual exercise (bilateral open-and-close actions) with MVF; bimanual exercise without MVF.	fMRI.	Bimanual exercise with MVF significantly increased the activity in precuneus and PCC, more than other conditions.

Szameitat et al. [56]	Five chronic right-hemispheric stroke patients (>one year after stroke), aged 57 to 67.	Unclear.	Action execution (left wrist flexion and extension), MI (MI of the same wrist movement), AO (watching a video showing the same action), passive movement and baseline.	fMRI.	AO activated right lateral medial anterior PMC and a small focus of right IPL than baseline result.

Wang et al. [57]	Five stroke patients (29 to 93 days after stroke), aged 53 to 72.	Severe arm paresis (a wrist extension ability of less than 20 degrees and metacarpophalangeal joint extension ability of less than 10 degrees).	Unilateral index finger-thumb opposition (by nonparetic hands) with virtual normal visual feedback or virtual MVF.	fMRI.	Four out of five patients displayed the lateralized activation toward the affected hemisphere (reflected by peak *T* values within the precuneus), evoked by virtual MVF.

Brunner et al. [47]	18 stroke patients, aged 41 to 79; first scan: 8.9 ± 4.1 days after stroke; second scan: 89.3 ± 8.3 days after stroke.	NHPT < 0.5 (pegs per second).	AO (a video of bimanual twisting of a cylindrical device) and its resting condition (a still image of the device being held); action execution (bimanual twisting of a cylindrical device) and its resting condition (hold the device without twisting).	fMRI.	AO (first scan): involvement of the occipital and temporal visual areas bilaterally with activation maxima in the MTG and ITG and occipital lobe. Patients also showed activation in the parietal and frontal areas and the IPL, SPL, IFG, and M1 were involved; AO (second scan): most activated clusters were observed in ITG and the ventral anterior of the thalamus, also in premotor areas, SMA and M1.

Dettmers et al. [48]	18 subcortical stroke patients (nine left stroke patients, aged 59.2 ± 7.1, 28.2 ± 40.9 months after stroke and nine right stroke patients aged 63 ± 10.3, 47.1 ± 89.5 months after stroke).	With the ability to grip a small object and release it by the paretic hand.	AO (static pictures) of object-related hand action by the paretic side, AO (movies) of object-related hand action by the paretic side, AO (same movies) with imagery (performing the shown action) and fixation.	fMRI.	AO (movies) elicited activation in visual cortex, SPL, prefrontal cortex, and superior and inferior frontal cortexes in both patient groups. AO (movies) with imagery revealed a very similar network as during AO (movies) alone.

Saleh et al. [55]	15 chronic stroke patients (>six months after stroke), aged 54 ± 12.	CMA: four to seven; CMH: three to seven.	Nonparetic hand action (finger flexion) with veridical feedback or MVF and the control (nonanthropomorphic objects).	fMRI (14 data).	MVF induced significant activation of the ipsilesional postcentral gyrus, M1, precuneus, contralesional postcentral gyrus, superior bank of the intraparietal sulcus and precuneus, and SMG. Connectivity between BA 1 and M1 and between BA 1 and S1 was significantly stronger after MVF.

Saleh et al. [34]	15 chronic stroke patients (>six months after stroke), aged 54 ± 12.	CMA: four to seven; CMH: three to seven.	Nonparetic hand action (finger flexion) with veridical feedback or MVF and the control (nonanthropomorphic objects).	fMRI (12 data).	MVF-induced activation of the ipsilesional primary motor cortex arose from the contralesional parietal cortex, in a region along the IPS.

Frenkel-Toledo et al. [49]	33 stroke patients aged 24 to 76, 23 to 132 days after stroke.	FMA zero to 66.	AO (reach and grasp action by left or right hands), observation of nonbiological videos and the eye close condition.	EEG.	AO induced mu suppression over SMC rather than observation of nonbiological videos; mu suppression was significantly diminished in the ipsilesional SMC (C3 or C4), compared with the contralesional SMC (C3 or C4); right IPL damage lowered mu suppression over the unaffected hemisphere.

Rossiter et al. [54]	10 stroke patients aged 56 ± 12, one to 114 months after stroke.	ARAT zero to 57.	Bilateral open-and-close hand action with MVF of nonparetic hand and bilateral open-and-close hand movement while viewing the paretic hand.	MEG.	Movement-related beta desynchronization was greater in contralesional compared to ipsilesional hemisphere. The asymmetry in movement-related beta desynchronization was more symmetrical in the condition with MVF.

Frenkel-Toledo et al. [13]	36 stroke patients aged 24 to 81 years, 23 to 132 days after stroke.	FMA zero to 66.	AO (reach and grasp action by left or right hands), observation of nonbiological videos, and the eye close condition.	EEG.	Failure to imitate correlated with diminished mu suppression in patients with IPL or IFG pars opercularis damage.

Kuk et al. [51]	20 chronic stroke patients (>six months after stroke); EG (*n* = 10): stroke patients aged 60.0 ± 9.36; CG (*n* = 10): stroke patients aged 59.70 ± 6.58.	With the ability to grasp a small cube (2.5 cm^3^) by the paretic hand.	EG: AO (watching videos of the actions of BBT performed by both hands), followed by performing the same task; CG: observation of nonbiological videos, followed by performing the same task.	EEG (*n* = 10, only for EG).	MTG was not activated after five sessions of AO, compared with pretraining.

Tani et al. [58]	11 stroke patients (18 to 1919 days after stroke), aged 64.1 ± 7.8.	Brunnstrom stage of the hand III–V.	AO (open-and-grasp action by the paretic hand), MI (the same actions by the paretic hand), and fixation.	EEG.	AO induced stronger mu suppression over the ipsilesional SMC (C3 or C4) than MI.

EG: experimental group; CG: control group; FMA-UE: Fugl-Meyer assessment upper extremity; AO: action observation; IFG: inferior frontal gyrus; SMG: supramarginal gyrus; fMRI: functional magnetic resonance imaging; MVF: mirror visual feedback; PCC: posterior cingular cortex; MI: motor imagery; PMC: premotor cortex; SMC: sensorimotor cortex; S1: primary somatosensory cortex; M1: primary motor cortex; NHPT: nine-hole peg test; MTG: middle temporal gyrus; ITG: inferior temporal gyrus; SPL: superior parietal lobe; IPL: inferior parietal lobe; PMd: dorsal premotor cortex; SMC: sensorimotor cortex; CMA: Chedokee-McMaster motor assessment arm scale; CMH: Chedokee-McMaster motor assessment hand scale; BA: Broadman area; EEG: electroencephalography; IPS: intraparietal sulcus; ARAT: action research arm test; MEG: magnetoencephalography; BBT: box and block test; ERD: event-related desynchronization.

**Table 4:** Methodological assessment of included studies using the PEDro scale^∗^.

Criterion	Ertelt et al. [22]	Michielsen et al. [52]	Bae et al. [45]	Sun et al. [28]
Eligibility criteria	Yes	Yes	No	Yes
Random allocation	1	1	1	1
Concealed allocation	0	1	0	0
Baseline comparability	1	1	1	1
Blind subjects	0	0	0	0
Blind therapists	0	0	0	0
Blind assessors	0	1	0	1
Adequate follow-up	1	1	0	1
Intention-to-treat analysis	0	1	0	1
Between group comparisons	1	1	1	1
Point estimates and variability	1	1	1	1
Total scores	5	8	4	7

^∗^The PEDro scores were taken from the PEDro website, except Ertelt et al. [22] and Sun et al. [28], which were rated by our team.

## Abbreviations

AO: Action observation
MVF: Mirror visual feedback
MNS: Mirror neuron system
fMRI: Functional magnetic resonance imaging
EEG: Electroencephalography
CIMT: Constraint-induced movement training
VR: Virtual reality
IFG: Inferior frontal gyrus
PMv: Ventral premotor cortex
IPL: Inferior parietal lobule
IPS: Intraparietal sulcus
MT: Mirror therapy
MEG: Magnetoencephalography
PET: Positron emission tomography
NIRS: Near-infrared spectrometry
ERD: Event-related desynchronization
DCM: Dynamic casual modelling
PMC: Premotor cortex
SMC: Sensorimotor cortex
MI: Motor imagery
STG: Superior temporal gyrus
SMA: Supplementary motor area
SMG: Supramarginal gyrus
PCC: Posterior cingulate cortex
MFG: Middle frontal gyrus.
